# Ability of *Muscidifurax raptorellus* and Other Parasitoids and Predators to Control *Drosophila suzukii* Populations in Raspberries in the Laboratory

**DOI:** 10.3390/insects10030068

**Published:** 2019-03-07

**Authors:** Phanie Bonneau, Justin Renkema, Valérie Fournier, Annabelle Firlej

**Affiliations:** 1 Centre de Recherche en Innovation sur les Végétaux (CRIV), Université Laval, Québec City, QC G1V 0A6, Canada; valerie.fournier@fsaa.ulaval.ca; 2 Institut de Recherche et de Développement en Agroenvironnement (IRDA), Saint-Bruno-de-Montarville, QC J3V 0G7, Canada; annabelle.firlej@irda.qc.ca; 3 London Research and Development Centre—Vineland Campus, Agriculture and Agri-Food Canada, Vineland Station, ON L0R 2E0, Canada; justin.renkema@canada.ca

**Keywords:** *Drosophila suzukii*, biological control, invasive pest, host-parasitoid interactions, predation

## Abstract

*Drosophila suzukii* is an invasive pest and economic threat to berry crops in Europe and the Americas. Current methods of control of this pest rely primarily on frequent applications of insecticides; therefore, there is a need for alternative control methods to reduce insecticide reliance. In this study, we evaluated the biological control potential of three parasitoid wasps: *Diglyphus isaea*, *Muscidifurax raptorellus* and *Pachycrepoideus vindemmiae*, and four predators: *Chrysoperla carnea*, *Dicyphus hesperus*, *Orius insidiosus* and *Podisus maculiventris*. Experiments were conducted for 15 days under controlled conditions in experimental arenas with *D. suzukii* females and raspberries, allowing for all life stages of *D. suzukii* to be available to natural enemies. Results showed the first evidence of *M. raptorellus*’s ability to parasitize *D. suzukii*, resulting in a 40% reduction. *Orius insidiosus*, *P. vindemmiae* and *C. carnea* were also efficient, reducing *D. suzukii* numbers by 49%, 43% and 32%, respectively. Predator preferences for each *D. suzukii* life stage were assessed. The clutch size, sex ratio and adult size variability of *D. suzukii* pupal parasitoids were also evaluated. This study expands the list of species that can effectively parasitize *D. suzukii* and provides new insights into the biological responses of *M. raptorellus* to *D. suzukii* pupae.

## 1. Introduction

*Drosophila suzukii* Matsumura (Diptera: Drosophilidae) is an invasive pest from Asia that is now considered a major economic threat to soft fruit crops in Europe and the Americas, causing up to 80% yield loss in some crops [1]. Unlike other vinegar flies which prefer decaying fruit, *D. suzukii* females damage ripening fruits by laying their eggs with a serrated ovipositor and most damage is caused by larvae feeding on the fruit pulp [2]. Raspberry, strawberry, blackberry, blueberry and cherry have been the main crops affected by *D. suzukii* since its invasion in 2008 in North America [1,3,4]. Raspberries are *D. suzukii*’s preferred host, with approximately $40 million in losses from 2009 to 2014 in California [5].

Synthetic insecticides are the primary control method for *D. suzukii* but repeated interventions may promote insect resistance and most products target only the adult stage, which is outside the fruit [6,7]. Spinosad (Entrust SC) is an effective biological insecticide for organic growers, but preharvest intervals and the number of allowable applications per season per crop make management difficult [8]. There are a few alternatives to chemical control: cultural control (sanitation, exclusion nets, mass trapping) and biological control [4,9,10,11].

Biological control may be an ecological, efficient and cost-effective strategy for organic growers, targeting *D. suzukii* life stages that are inaccessible by insecticides, without generating development of resistance. Indigenous natural enemies are likely present and may play a role in reducing *D. suzukii* in both cropped and uncultivated habitats, hence the crucial importance of conservation of natural enemies’ biodiversity [11,12,13]. Reducing the use of synthetic chemical insecticides and promoting integrated pest management (IPM) approaches may contribute to the preservation of natural enemies’ richness and activity [14]. However, these beneficial insects might not be present in adequate numbers to provide effective control of *D. suzukii* in high value fruit crops with a low economic injury level [5,11]. Artificially multiplying effective beneficial insects and releasing them en masse would increase their numbers and activity in fruit crop ecosystems but require farmers to use environmentally safe and compatible external inputs [15]. Predators and parasitoids are commonly used as biological control agents and, while parasitoids are mostly specific and have a high self-propagating capacity, predators are more generalist and have the ability to actively capture and suppress their prey without delay [15]. Therefore, augmentative releases of predators and parasitoids may be a relevant strategy to reduce *D. suzukii* populations, not only in fruit crops, but also in closely adjacent vegetation [16]. Consequently, methods for the augmentative releases of natural enemies against *D. suzukii* need more research before recommendations can be made.

Research on classical biological control of *D. suzukii* has shown that larval endoparasitoids *Asobara japonica* Belokobylskij (Hymenoptera: Braconidae), *Ganaspis brasiliensis* Ihering and *Leptopilina japonica japonica* Novkovic and Kimura (Hymenoptera: Figitidae) from *D. suzukii*’s native range can develop successfully from *D. suzukii* [17]. However, satisfying regulations to avoid non-target effects on native species and the ecosystem make the importation of exotic biological control agents an onerous and lengthy procedure [18]. Meanwhile, native natural enemies such as parasitoids and predators that are already in the invaded area of *D. suzukii* may be able to adapt and would provide a prompter solution than classical biological control agents.

In North America and Europe, studies on native pupal ectoparasitoids demonstrated that *Pachycrepoideus vindemmiae* Rondani (Hymenoptera: Pteromalidae) and *Trichopria drosophilae* Perkins (Hymenoptera: Diapriidae) parasitized 53%–60% and 38%–76%, respectively, of *D. suzukii* in the laboratory [19,20]. However, there are few studies of other parasitoids native to North America that may be able to use *D. suzukii* pupae as hosts [21], and the majority of parasitoids tested against *D. suzukii* are not currently commercially available. Therefore, it is crucial to find other successful native parasitoids readily available to berry growers in North America.

In parallel, North American and European studies on *Orius* spp. (Heteroptera: Anthocoridae), *Anthocoris nemoralis* Fabricius (Heteroptera: Anthocoridae), *Dalotia coriaria* Kraatz (Coleoptera: Staphylinidae) and *Labidura riparia* Pallas (Dermaptera: Labiduridae) confirmed that these predators can feed on *D. suzukii* larval, pupal or adult life stages [22,23,24,25]. However, predator efficacy for *D. suzukii* control has been highly variable in these studies, and few have investigated egg predation [26,27].

In order to reduce insecticide reliance, the primary goal of this study was to evaluate commercially-available natural enemies not previously tested against *D. suzukii* and to test their effectiveness with a life-stage specific experiment (for some predators) and when simultaneously presented with multiple *D. suzukii* life stages in infested raspberries. Therefore, a suite of available natural enemies for eventual augmentative releases including two parasitoid wasps and four insect predators was chosen from distributors in Canada. 

The selected parasitoid wasps were a pupal ectoparasitoid, *Muscidifurax raptorellus* Kogan and Legner (Hymenoptera: Pteromalidae) and a larval ectoparasitoid *Diglyphus isaea* Walker (Hymenoptera: Eulophidae). The gregarious idiobiont *M. raptorellus*, a filth fly parasite commonly used in augmentative biological control in livestock operations, was chosen because of its wide range of potential dipteran hosts under a large scope of production systems and in many different geographic areas [28,29]. Gregarious *D. isaea* parasitizes a wide variety of dipteran leafminers and was selected for its ability to inspect the plant to find hidden fly larvae [30]. For comparison purposes, the solitary *P. vindemmiae* was included and the parasitoid clutch size, sex ratio and size variability of successfully emerged adults were recorded.

The selected generalist predators were *Podisus maculiventris* Say (Hemiptera: Pentatomidae), *Dicyphus hesperus* Knight (Heteroptera: Miridae) and larval *Chrysoperla carnea* Stephens (Neuroptera: Chrysopidae). The predatory shield bug *P. maculiventris* was chosen because it attacks the eggs, larvae and adults of more than 90 arthropod species spread over eight orders [31]. The polyphagous predatory mirid *D. hesperus* occurs in a wide range of greenhouse crops, locates prey easily [32] and was selected because a related species, *Dicyphus tamaninii*, has been found in *D. suzukii*-infested fruit [33]. Larval *C. carnea*, which is highly voracious, can consume 50 prey per day [34] and was selected for its wide host range, hunting capacity and resistance to certain pesticides [35]. None of these species have been evaluated against *D. suzukii* [26,27]. For comparison purposes, *Orius insidiosus* Say (Heteroptera: Anthocoridae) was included because it successfully caused 12–50% larval *D. suzukii* mortality in the laboratory [25].

## 2. Materials and Methods 

### 2.1. Insect Sources

*Drosophila suzukii* was obtained from a colony maintained at 23 ± 1 °C; 16 L: 8 D; 50 ± 10% RH in plexiglass cages in the Institut de recherche et de développement en agroenvironnement (IRDA) in Saint-Bruno-de-Montarville, Quebec. The colony was started in summer 2014 from infested blueberry collected near St-Jude, Quebec. Eggs were laid in raspberries placed directly on the diet, in which larvae migrated. The diet consisted of a mixture of 70 g of brewer’s yeast, 150 g of carrot powder, 2.5 g of sodium benzoate, 2 g of methylparaben, 50 mL of 1 N HCl and 800 mL of distilled water. Flies were also provided with a mixture of white sugar and baker’s yeast (1:1) in a Petri dish and water on a dental cotton roll.

The parasitoid wasp *M. raptorellus* was purchased from Bugs for Bugs (Guelph, ON, Canada). The parasitoid was sold as a mix in pupae of *Musca domestica* Linnaeus (Diptera: Muscidae), from which three parasitoid wasp species (Pteromalidae) emerge. Emerged parasitoids were sorted and male and female *M. raptorellus* were transferred using an insect vacuum to a separate plexiglass cage. Prior to use, parasitoids were provided with honey and water (1:1) on a dental cotton roll.

The parasitoid wasp *P. vindemmiae* was from a colony started in summer 2017 and maintained at 22 ± 1 °C; 16 L: 8 D; 55 ± 10% RH in a plexiglass cage in IRDA’s laboratory. The colony was reared on *D. suzukii* pupae and originated from a *P. vindemmiae* colony at Agriculture and Agri-Food Canada (AAFC) in Agassiz, British Columbia, which was started from infested cherries collected near Summerland, British Columbia (BC), in 2015. 

The larval predator *C. carnea*, the adult predator *O. insidiosus* and the parasitoid wasp *D. isaea* were purchased from PlantProducts (Leamington, ON, Canada) and kept in their shipping package with an ice pack for one day prior to use. 

The adult predator *D. hesperus* and the nymph predator *P. maculiventris* were purchased from Anatis Bioprotection (Saint-Jacques-le-Mineur, QC, Canada) and kept in their shipping package with an ice pack for one day prior to use. 

### 2.2. Parasitoid and Predator Experiment with Multiple D. suzukii Life Stages

The experimental arena was a clear plastic box (1 L) with a 40-cm length mesh sleeve with an opening on one end. Each arena contained five store-purchased red raspberries, washed in water, and placed in a clear plastic dish (177 mL, 10-cm diameter) with a cotton square to absorb excess liquid during the experiment.

On day 1 of the experiments, five 3-day old *D. suzukii* mated adult females were placed in each plastic box to lay eggs in raspberries (22 ± 1 °C; 16 L: 8 D; 55 ± 10% RH). Two raspberries were added to each plastic box every three days throughout the experiment in order to avoid the presence of decaying egg laying sites.

Eighteen females of each parasitoid species (3 to 5 days old and previously mated) or 18 individuals of each predator species were placed in arenas (one species per arena) on day 4 for larval parasitoids, on day 9 for pupal parasitoids and on day 3 for predators of the experiment, to ensure larvae and/or pupae were available. Eighteen parasitoids or predators were used based on the results of a pre-test with 12, 18 or 24 individuals and the densities used in a related study [36].

The experiments were conducted in two blocks, with the three parasitoid species and a control in the first and the four predator species and a control in the second. Each parasitoid and predator species was replicated eight times. After 15 days (time required to complete one *D. suzukii* life cycle under optimal laboratory conditions), the live predators, live parasitoids, *D. suzukii* larvae, pupae and live and dead adults were counted. The clear plastic dish with raspberries was filled with water so that *D. suzukii* larvae and pupae floated and could easily be removed and counted. The design of this experiment allowed age structure to be established so natural enemies were in the presence of all life stages of *D. suzukii* (egg, larva, pupa, adult) in each arena at the same time and the control (no natural enemies) was used as the base for comparisons among treatments.

For arenas with larval parasitoids, recovered *D. suzukii* larvae were placed in a 500-mL plastic container with a lid containing fine mesh for ventilation, filled with drosophila diet and kept in growth chambers for 35 days to determine the *D. suzukii* pupation rate or emergence rate of adult parasitoids. For arenas with pupal parasitoids, *D. suzukii* pupae recovered were placed singly in wells of an ELISA plate and kept in growth chambers for 35 days to determine the emergence rates of adult parasitoids or *D. suzukii*. 

The success rate of parasitism (parasitoid adult emergence), the clutch size (number of parasitoids from one pupa), sex ratio and adult size variability were determined. The last three parameters are used in biological control studies to predict the efficacy of insect parasitoids and their foraging behavior [37]. While the clutch size and sex ratio are foraging decisions based on host quality, the progeny body size, directly influenced by the clutch size, is a direct indicator of fitness [37].

The adult parasitoid size was estimated by measuring the length (mm) of the left hind tibia [38]. For *M. raptorellus*, the measurements of 15 males and 15 females that emerged from *M. domestica* pupae served as a control. For individuals from *D. suzukii* pupae recovered in the 15-day experiment, 15 males and 15 females were measured when there was one parasitoid per pupa and 15 pupae were randomly selected when there were two or more parasitoids per pupa. For *P. vindemmiae*, the measurements of 15 males and 15 females that emerged from *D. suzukii* pupae in the colony served as a control. For individuals from *D. suzukii* pupae recovered in the experiment, 15 males and 15 females were measured, as *P. vindemmiae* is a solitary pupal parasitoid. The measurements were made by removing the hind tibia from the body of the insect, photographing and measuring it with an electronic stereomicroscope, Zeiss SteREO Discovery V12 with Zeiss Axiocam 503 3 Mpix video camera, using software, ZEN 2012 SP2 blue edition (© Carl Zeiss Microscopy GmbH, 2011).

### 2.3. Predator Experiment with Single D. suzukii Life Stage

The experimental arena was a clear, plastic container with a lid (177 mL, 10-cm diameter) and a raspberry leaf (cv. ‘Polana’) from unsprayed research plots that was laid flat and had its petiole inserted into a water-filled 2-mL microcentrifuge tube. Each arena contained six individuals of one *D. suzukii* life stage (eggs, larvae, pupae or adults) which were placed on top of the leaf which served as a support. Eggs were collected from a Petri dish filled with store-purchased raspberry jam that was placed in a *D. suzukii* colony cage for two hours. Eggs were rinsed with distilled water then placed on the central vein of the leaf with a paintbrush. Larvae and pupae were collected from the artificial diet used in the colony cage, rinsed with distilled water and placed onto leaves with a paintbrush. Adults were collected from the emerged pupae that were isolated in a cage to ensure that the flies were all five days old and three males and three females were placed into the experimental arenas using an insect vacuum. A predator (either adult *O. insidiosus*, adult *D. hesperus*, nymph *P. maculiventris* or larval *C. carnea*), starved for 24 h, was placed in each arena. Arenas were held at 22 ± 1 °C; 16 L: 8 D; 55 ± 10% RH in a growth chamber. No food was provided to *D. suzukii* adults or larvae to prevent predators from feeding on it. After 24 h, predators were removed and the number of live *D. suzukii* individuals for each life stage was recovered from the leaves except for the pupae which were held seven additional days for adult emergence. There were 15 replications of each predator species and a control without a predator, for each of the four *D. suzukii* life stages.

### 2.4. Data Analysis

Data were tested with the Shapiro–Wilk test for normality and error variance for homoscedasticity, and analyses were conducted using JMP software (version 12.0.1, SAS Institute Inc., Cary, NC, USA). Data from the parasitoid and predator experiments with multiple *D. suzukii* life stages were analyzed using a one-way analysis of variance (ANOVA), followed by Tukey–Kramer’s HSD (*p* < 0.05) for post-hoc comparisons effects of natural enemy species on the multiple *D. suzukii* life stages, except for *D. suzukii* live adult data, which were non-parametric and analyzed using a Kruskal–Wallis test, followed by post-hoc Dunn’s test (*p* < 0.05). Data from the predator experiments with a single *D. suzukii* life stage were non-parametric and analyzed using a Kruskal–Wallis test, followed by post-hoc Dunn’s test (*p* < 0.05) to compare means of live *D. suzukii* among predators. Data from parasitized *D. suzukii* pupae for each pupal parasitoid wasp species were analyzed using a Student’s *t*-test (*p* < 0.05) to compare the parasitism rates. Data from individual parasitoid wasps that emerged from *D. suzukii* pupae were analyzed using a Chi-square test (*p* < 0.05) to compare male: female ratios for each clutch size. The leg measurements of *M. raptorellus* that emerged from *D. suzukii* pupae were non-parametric and analyzed using a Kruskal–Wallis test, followed by a post-hoc Dunn’s test (*p* < 0.05). However, the leg measurements of individuals that emerged from *P. vindemmiae* were normally distributed and analyzed using a Student’s *t*-test (*p* < 0.05). 

## 3. Results

### 3.1. Parasitoid and Predator Experiment with Multiple D. suzukii Life Stages

**Figure 1:**
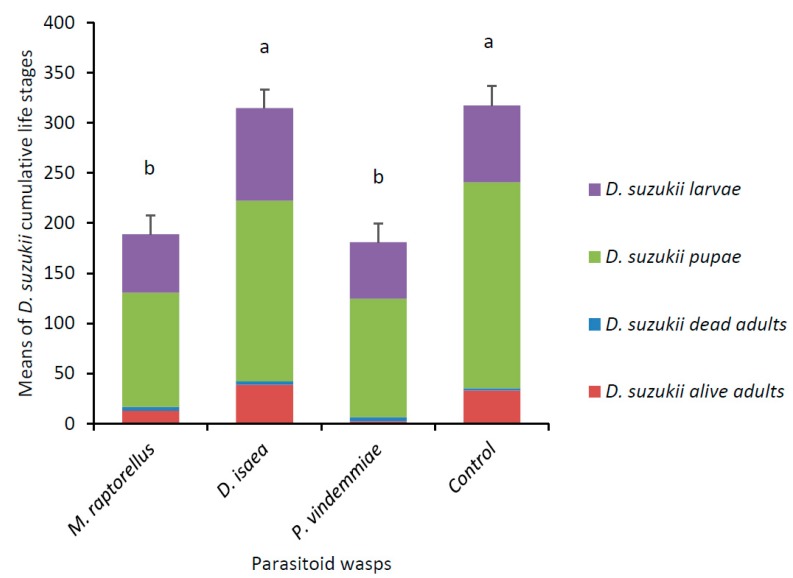
Means (± SE) of *D. suzukii* cumulative life stages (larvae, pupae, and dead and alive adults) recovered after 15 days when exposed to female parasitoid wasps. Each arena (*n* = 8 per parasitoid wasp species) contained five *D. suzukii* mated females and raspberries at the beginning of the experiment, with 18 larval parasitoids added on day 4 and 18 pupal parasitoids added on day 9. Cumulative means with the same letter were not significantly different (Tukey’s HSD, *p* < 0.05).

At the end of the 15-day experiment, *M. raptorellus* and *P. vindemmiae* significantly reduced *D. suzukii* numbers by 40% and 43%, respectively compared to the control (F_3,27_ = 15.97, *p* < 0.0001) (Figure 1). Only *P. vindemmiae* caused a decrease in the mean number of *D. suzukii* live adults (χ^2^ = 25.54, df = 3, *p* < 0.0001). Both pupal parasitoids decreased the number of *D. suzukii* pupae (F_3,27_ = 8.81, *p* = 0.0003). Larval parasitoid *D. isaea* did not have an impact on any of the *D. suzukii* life stages. At the end of the experiment, there were 7.0 ± 4.2 live *M. raptorellus* and 14.3 ± 2.7 live *P. vindemmiae* (mean ± SD), but all individuals of *D. isaea* were dead.

**Figure 2:**
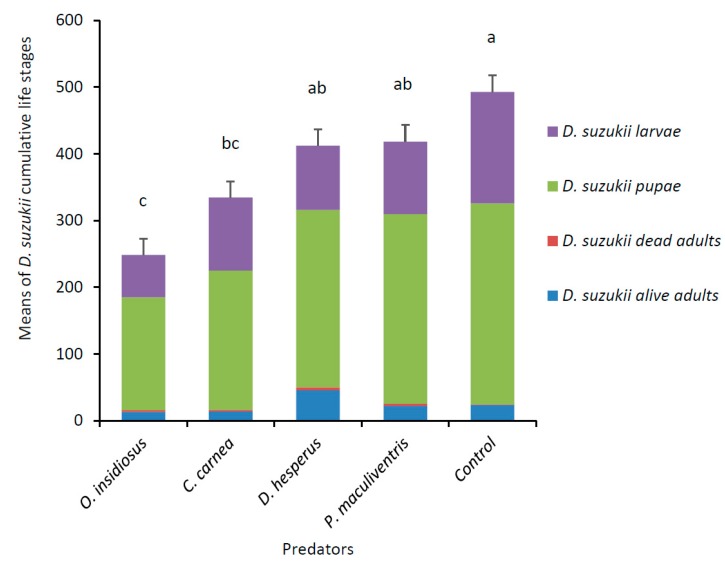
Means (± SE) of *D. suzukii* cumulative life stages (larvae, pupae, and dead and alive adults) recovered after 15 days when exposed to predators. Each arena (*n* = 8 per predator species) contained five *D. suzukii* mated females and raspberries at the beginning of the experiment, with 18 predators added on day 3. Cumulative means with the same letter were not significantly different (Tukey’s HSD, *p* < 0.05).

After 15 days, there was a significant effect of predator species on the total number of all *D. suzukii* life stages recovered (F_4,33_ = 14.01, *p* < 0.0001). *Orius insidiosus* adults and *C. carnea* larvae reduced *D. suzukii* numbers by 49% and 32%, respectively, whereas *D. hesperus* and *P. maculiventris* did not significantly reduce *D. suzukii* cumulative life stages compared to the control (Figure 2). Predators *O. insidiosus* and *C. carnea* caused a decrease in the number of *D. suzukii* live adults (χ^2^ = 20.55, df = 4, *p* = 0.0004) and pupae (F_4,33_ = 6.32, *p* = 0.0007). Predators *O. insidiosus* and *D. hesperus* decreased the number of *D. suzukii* larvae (F_4,33_ = 6.91, *p* = 0.0004). Predator *P. maculiventris* did not have an impact on any of the *D. suzukii* life stages. At the end of the experiment, 4.5 ± 3.5 *O. insidiosus*, 2.4 ± 1.5 *C. carnea*, 13.0 ± 0.9 *D. hesperus* and 7.6 ± 1.7 *P. maculiventris* (mean ± SD) were recovered alive.

### 3.2. Predator Experiment with Single D. suzukii Life Stage

**Figure 3:**
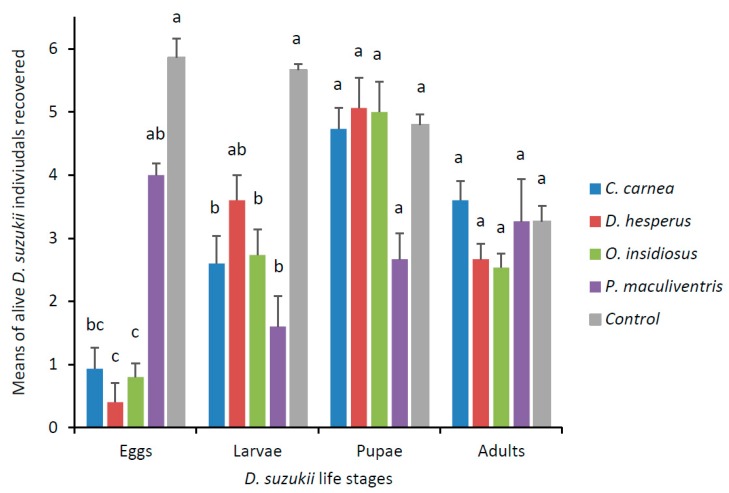
Mean (± SEM) number of *D. suzukii* life stages recovered after 24-h exposure to predators (either larval *C. carnea*, adult *D. hesperus*, adult *O. insidiosus* or nymph *P. maculiventris*). Each arena (*n* = 15 per predator species) contained six *D. suzukii* (either eggs, larvae, pupae or adults) and one predator. Means for each life stages with the same letter were not significantly different (Kruskal–Wallis post-hoc Dunn’s test, *p* < 0.05).

Predation occurred primarily on *D. suzukii* eggs and larvae (Figure 3). Predators *C. carnea, D. hesperus* and *O. insidiosus* were able to feed on *D. suzukii* eggs (χ^2^ = 47.88, df = 4, *p* < 0.0001) and predators *C. carnea*, *O. insidiosus* and *P. maculiventris* were able to feed on *D. suzukii* larvae (χ^2^ = 34.54, df = 4, *p* < 0.0001). None of the four predators were able to predate significantly *D. suzukii* adults and pupae (χ^2^ = 10.25, df = 4, *p* = 0.0363; χ^2^ = 8.54, df = 4, *p* = 0.0737). At the end of the experiment, all predators were found alive except for two *D. hesperus*. Most of the different life stages were recovered alive in the control treatments except for adults, nearly 50% of which died in the absence of food.

### 3.3. Pupal Parasitoid Observations

**Figure 4:**
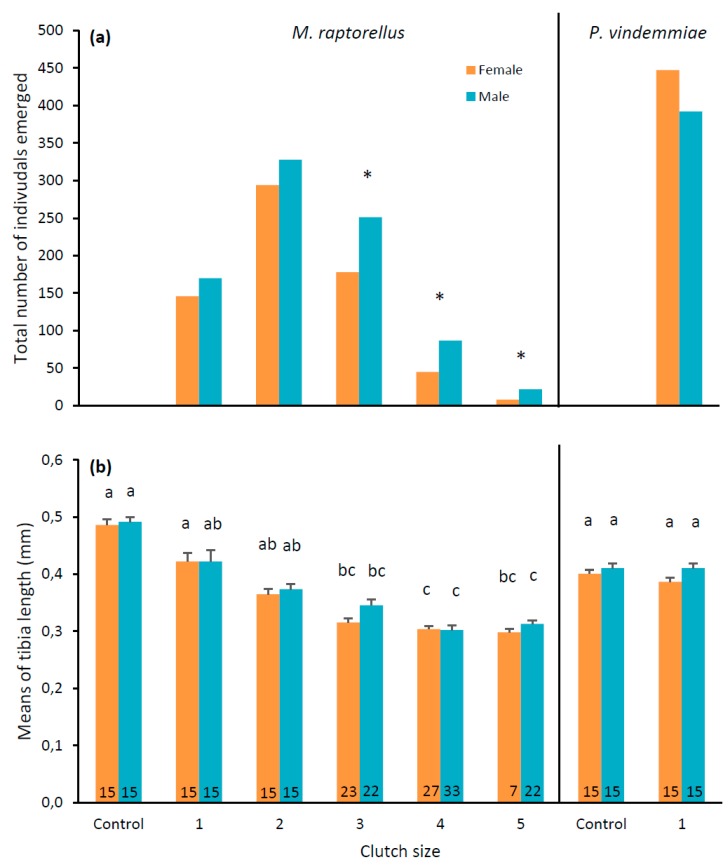
Total number of individuals (**a**) and means (± SE) of left hind tibia length (**b**) of *M. raptorellus* and *P. vindemmiae* parasitoids that emerged from *D. suzukii* pupae at various clutch sizes in raspberries under laboratory conditions. Controls were *M. raptorellus* that emerged from *M. domestica* pupae and *P. vindemmiae* that emerged from *D. suzukii* pupae used in the rearing colony. Asterisks above bars in panel (**a**) indicate male: female ratios different than 1:1 for each clutch size (Chi-square, *p* < 0.05), and bars with the same letter in panel (**b**) are not significantly different (*M. raptorellus*: Kruskal–Wallis post-hoc Dunn’s test *p* < 0.05; *P. vindemmiae*: Student’s *t*-test, *p* < 0.05) for each sex and parasitoid species. Numbers in bars in panel (**b**) are the individuals measured.

*Muscidifurax raptorellus* and *P. vindemmiae* both successfully parasitized *D. suzukii* pupae and had similar parasitism rates (t_14_ = 0.28, *p* = 0.7804) (Table 1). There was a total of 810 parasitized *D. suzukii* pupae by *M. raptorellus* after 15 days out of 1866 in total, which produced an average of 1.9 parasitoid offspring (861 males and 674 females) from *D. suzukii* with an average of 43.9% females (Figure 4a). For *P. vindemmiae*, there was a total of 839 parasitized *D. suzukii* pupae after 15 days out of 1873 in total, which produced an average of one parasitoid offspring (392 males and 447 females) from *D. suzukii* with an average of 53.5% females (Figure 4a). For *M. raptorellus*, the percentage of female progeny decreased when the clutch size increased and the sex ratio of the clutch size containing three (41.5% female), four (34.1% female) and five (26.7% female) parasitoids was significantly different than 1:1 (χ^2^ = 12.42, df = 1, *p* = 0.0004; χ^2^ = 13.36, df = 1, *p* = 0.0003; χ^2^ = 6.53, df = 1, *p* = 0.0106, respectively) (Figure 4a). As for *P. vindemmiae*, the sex ratio was not significantly different from 1:1 (χ^2^ = 3.61, df = 1, *p* = 0.0576) (Figure 4a). There was one pupa from which six *M. raptorellus* adults (three males and three females) emerged that is not shown in the figure. The size of *M. raptorellus* from *M. domestica* pupae (control) is larger than from *D. suzukii* pupae but significantly different only for clutch sizes 3, 4 and 5, for both males and females (χ^2^ = 68.96, df = 5, *p* < 0.0001; χ^2^ = 67.23, df = 5, *p* < 0.0001, respectively) (Figure 4b). The size of *P. vindemmiae* from *D. suzukii* pupae in the experiment is not significantly different from the control, for both males and females (t_28_ = 0.00, *p* = 0.9971; t_28_ = 1.36, *p* = 0.1848, respectively) (Figure 4b).

## 4. Discussion

Our results provided the first evidence of *M. raptorellus*’s ability to parasitize *D. suzukii* pupae in the laboratory and demonstrated a level of efficacy comparable to *P. vindemmiae*. As with *P. vindemmiae*, *M. raptorellus* females were able to lay viable eggs in *D. suzukii* pupae and larvae readily fed on *D. suzukii* pupae, resulting in successful adult emergence. Both pupal parasitoid species also showed the same success rates of parasitism, although *P. vindemmiae*’s rate in our study (45%) is lower than that reported in Europe (57% and 53%) [20,39]. 

*Diglyphus isaea* was not able to parasitize *D. suzukii*, as no parasitoids emerged from *D. suzukii* larvae kept in growth chambers and most *D. suzukii* pupated successfully. We also observed no parasitoid adults alive in experimental arenas at the end of the experiment. Cheah and Coaker [40] reported that *D. isaea* females ignored live host larvae of *Chromatomyia syngenesiae* isolated from their mines, suggesting that the host-finding process required host plant stimuli. *Diglyphus isaea* is primarily a parasitoid of leafmining insects [30] and probably would simply not recognize and assess *D. suzukii* larvae at the surface of fruits as potential hosts [41]. 

**Figure 5:**
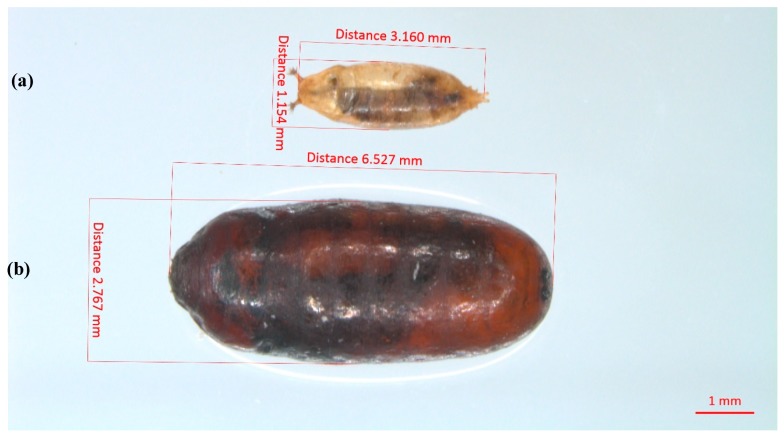
Size differences between host *D. suzukii* and *M. domestica* pupae. (**a**) parasitized *D. suzukii* pupa; (**b**) parasitized *M. domestica* pupa.

Up to six gregarious *M. raptorellus* adults survived and emerged from a single *D. suzukii* pupa in this experiment, while Harvey et al. [42] reported up to 15 adults emerging from a single *M. domestica* pupa. However, the clutch size frequency decreased as the number of individuals emerging per host increased for both host species [42]. The most common clutch sizes in *D. suzukii* were 1–3 eggs, which was the same as in *M. domestica* [42]. Similarly, Petersen and Currey [29] noted that *M. raptorellus* laid 2–3 eggs per host when there was a high availability of *M. domestica*. Geden and Moon [43] reported that *M. raptorellus* produced 2.2–2.9 progeny per parasitized host from five dipteran hosts of different sizes, comparable to the production of an average of 1.9 parasitoids per *D. suzukii* pupae in this study. It appears that *M. raptorellus* does not strongly discriminate hosts for quality and does not adjust the clutch size according to the host size, knowing that *D. suzukii* pupae are more than ten times smaller than *M. domestica* pupae (Figure 5). 

The clutch size influenced the parasitoid progeny size, as reported in Harvey et al. [42], and an *M. raptorellus* clutch size of three or more eggs, adult female and male wasp size decreased with *D. suzukii* as the host. Smaller female wasps may result in reduced fitness, as the ability to control dispersal and lifetime egg production and reproductive success may be reduced [38]. However, as there was no significant difference between the sizes of *M. raptorellus* that emerged from *M. domestica* (control) and from *D. suzukii* (1–2 eggs per host), *D. suzukii*, as the host, may not cause reduced fitness for most of the *M. raptorellus* progeny.

However, regardless of the clutch size (1–5), we observed high variability in the size of *M. raptorellus* siblings of both sexes within the same pupae. Furthermore, most pupae with a single emergent parasitoid also contained small dead larvae of non-emerged parasitoids, which would explain the different sizes of singly emerged adults. Size variability, irrespective of the clutch size, is typical in gregarious idiobiont parasitoids, as many offspring can develop inside a single host [44]. For idiobiont species such as *M. raptorellus* that paralyze their host, host development is stopped at oviposition and the resources are then immutable, as the amount of food available inside the pupae does not increase [28,38]. Therefore, space and resources are limited, and competition increases as more parasitoid larvae feed on the host. 

The low female ratio of *M. raptorellus* from *D. suzukii* hosts may be explained by the general sex-allocation theory that parasitoid wasps will invest in female offspring in larger hosts and use male eggs for less-than-optimal hosts [41,45]. Harvey and Gols [46] reported that *M. raptorellus* had 60% female progeny in *M. domestica* host and 76% female progeny in a larger host, *Calliphora vomitoria* Linnaeus, and as the clutch size increased, the percentage of female progeny increased in the latter host from 52% with a clutch size of one and two eggs to 81% with a clutch size of five eggs or more. We observed the opposite trend in this experiment with the smaller *D. suzukii* host—as the clutch size increased, the percentage of female progeny decreased from 44% to 27%. However, as there was no significant difference from a 1:1 sex ratio of *M. raptorellus* progeny from *D. suzukii* when the clutch size was 1–2 eggs, *D. suzukii* pupa may be a suitable host for most of the *M. raptorellus* progeny.

As expected, the solitary parasitoid *P. vindemmiae* produced one parasitoid per *D. suzukii* pupa in the control and experiment. The sex ratio of progeny was not different from 1:1, and the percentage of female progeny (53%) was similar to the parent colony at AAFC Agassiz [47].

Idiobiont ectoparasitoids such as *M. raptorellus* and *P. vindemmiae* are often associated with concealed hosts [14]. Therefore, *D. suzukii* pupae hidden among decomposed raspberries inside the experimental arenas may represent a suitable host for these parasitoids. Both ectoparasitoid species tend to be more adaptable in their biological response to different hosts and are likely to search for a specific type of habitat or host location instead of a specific type of host itself [14]. This suggests that *M. raptorellus* may be a good pupal parasitoid of *D. suzukii* pupae fallen from fruits and buried in the soil.

The results from our predator experiments with single and multiple *D. suzukii* life stages showed that *O. insidiosus* and *C. carnea* were efficient *D. suzukii* predators. By conducting a, no-choice experiment in a simple environment, we determined which easily accessible *D. suzukii* life stages were attacked by selected predators. We found that *P. maculiventris* fed only on larvae, and *D. hesperus* fed only on eggs, while *C. carnea* and *O. insidiosus* both fed on *D. suzukii* eggs and larvae. None of the predators appeared to be able to attack pupal or adult *D. suzukii*, but we observed opportunistic feeding by some predators on adults that were weak or dead. 

Prey finding and acceptance can be affected by predator hunting and mobility capacities, nutritional benefits and the chemical composition of the prey cuticle [41]. Though destroyed *D. suzukii* pupal cases were found in experimental arenas, pupal cuticles may be impenetrable by predatory bugs and lacewings with piercing-sucking mouthparts. Nearly all predators mentioned in studies that report predation of *D. suzukii* pupae have chewing mouthparts [23,48,49]. Adult flies may be difficult for predators to capture in small experimental arenas where flying insects are too confined. Cuthbertson et al. [22] reported significant predation on adult flies by *Orius* spp. in a small experimental arena but only after 72 h. We also observed that *D. suzukii* eggs may be too small for *P. maculiventris* which possess a large proboscis to stab and carry their prey.

By conducting a more complex experiment with all *D. suzukii* life stages simultaneously present in raspberries, we determined predator efficiency that mimics field situations. *Podisus maculiventris* and *D. hesperus* reduced *D. suzukii* numbers by 15% and 16% respectively, but suppression was not significantly different from the control. It is likely that these two predators were not able to prevent exponential *D. suzukii* population growth because they only prey on one life stage, as shown in the no-choice trial. In contrast, *C. carnea* and *O. insidiosus* reduced *D. suzukii* numbers by 32% and 49%, respectively. The efficacy of *C. carnea* and *O. insidiosus* in a more complex environment may be explained by their ability to prey on two *D. suzukii* life stages, as shown in the no-choice trial. Therefore, it appears that predators that feed primarily on early *D. suzukii* life stages, such as eggs and larvae, can suppress *D. suzukii* population increases inside the experimental arena over the course of the experiment, while those that are only capable of consuming one life stage are less effective. The presence of raspberries in the experimental arenas may also have played a role in the efficacy of the predators and their foraging behavior. We observed *O. insidiosus* and *C. carnea* foraging on the surface of fruits for *D. suzukii* eggs and larvae, as was reported for *O. insidiosus* in Woltz et al. [25]. As the raspberries started to decompose, all the predators gained easier access to *D. suzukii* life stages. In addition, the suppression of *D. suzukii* may also be attributable to nonconsumptive predator effects [50]. Predators may have physically disturbed *D. suzukii* females during oviposition which would have resulted in reduced *D. suzukii* numbers.

## 5. Conclusions

Our study provided new additional information on the host range of the pupal parasitoid *M. raptorellus* and its ability to parasitize invasive *D. suzukii*. *Muscidifurax raptorellus* had similar parasitism rates to *P. vindemmiae*, and both reduced *D. suzukii* numbers in raspberries in the laboratory by 40 and 43%, respectively. Studies have shown that *P. vindemmiae* parasitized *D. suzukii* in the laboratory and field. However, in a natural environment, *P. vindemmiae* was not sufficiently abundant to prevent exponential *D. suzukii* population growth [39,51,52]. As a potential solution, *M. raptorellus* is commercially available in Canada and the United States and could be released in sufficient numbers to suppress *D. suzukii*. Further study on the growth and development of *M. raptorellus* in *D. suzukii* pupae is needed to better understand the host–parasitoid interactions.

Our results also confirmed that predatory natural enemies, *O. insidiosus* and *C. carnea*, were effective at reducing *D. suzukii* by almost 50%. In conclusion, all four of the effective natural enemies are present in agroecosystems in North America, were complementary in the life stages of *D. suzukii* they attacked and may play a role in the suppression of *D. suzukii* in fruit crop fields. Further research is needed to precisely measure the combined impacts of multiple natural enemies under field conditions, to evaluate a possible preference for alternative preys or hosts, and to optimize and support the augmentative releases of commercially-available species.

## Figures and Tables

**Table 1:** Successful parasitism for each pupal parasitoid wasp species (*n* = 18) when exposed to *D. suzukii* developing in raspberries in the laboratory. Mean numbers of parasitized pupae with the same letter were not significantly different (Student’s *t*-test, *p* < 0.05).

Parasitoid Species	Mean Number of *D. suzukii* Pupae per Repetition (± SD)	Mean Number of Parasitized *D. suzukii* Pupae per Repetition with Parasitoid Emergence (± SD)	Successful Parasitism (%)
***M. raptorellus***	232.3 ± 50.9	100.9 ± 26.1 a	43.4
***P. vindemmiae***	232.5 ± 45.5	104.3 ± 21.2 a	44.8
